# Supplementary material for: Patterns of Intron Gain and Loss in Fungi
Source: PLoS Biol. 2004 Nov 30;2(12):e422. doi: 10.1371/journal.pbio.0020422 (PMC532390; doi:10.1371/journal.pbio.0020422)
Supplement: Table S1 — Also available at http://genes.mit.edu/NielsenEtAl/. (4.3 MB ZIP). [file pbio.0020422.st001.zip › NielsenEtAl/html/1250.html]

AN4589.1.NCU04127.1.MG02742.1.FG08871.1


```
 CLUSTAL W (1.82) Multiple Sequence Alignments - Introns Inserted


Sequence 1: MG02742.1	70 aa
Sequence 2: FG08871.1	70 aa
Sequence 3: NCU04127.1	70 aa
Sequence 4: AN4589.1	70 aa
Alignment Length: 70 aa
Number Identitical Residues: 39 aa
Alignment Score (without introns) 2010


MG02742.1 	MSDSVQEILDVPREFVKDGIQFLNRCQKP1DKKEFRQICQAVGVGFLI~MG~AVGYIVKL
NCU04127.1	MADQIQEILDVPREFLKDGIQFIKKCQKP1DRREFIKISQAVGTGFLI1MG0AVGYLVKL
FG08871.1 	MSDQVQEILEVPSEFVRDGVQFMRRCTKP1DQKEFLRLCQAVGVGFLI~MG~AVGYVVKL
AN4589.1  	MSEQVQELLNIPQEFLRDGMQFVNRSQKP1DKREFIKISQAVGTGFLI~MG~FIGYIVKL
          	*::.:**:*::* **::**:**:.:. ** *::** ::.****.**** **  :**:***

MG02742.1 	V1HIPLNNILVGGA
NCU04127.1	I1HIPLNQVLVGGA
FG08871.1 	V1HIPLNHALVGSA
AN4589.1  	I1HIPVNNVLVGGA
          	: ***:*: ***.*
```
